# Development and validation of a diagnostic model for tuberculous meningitis based on laboratory data

**DOI:** 10.3389/fcimb.2025.1579827

**Published:** 2025-05-20

**Authors:** Fuyong Liu, Zheng Li, Xuejiao Li, Wei Hong, Yanlin Zhou, Yungang Han, Shuang Xia, Jiao Tan, Yunchang Yang, Shiqi Li, Zhi Li, Wenyi He, Huihui Chen, Pengxiang Li, Yali Wang, Xu Yang, Jingcai Gao, Wei Wang

**Affiliations:** ^1^ School of Basic Medical Sciences, North Henan Medical University, Xinxiang, China; ^2^ Department of Medical Laboratory, Henan Provincial Chest Hospital, Zhengzhou, China

**Keywords:** tuberculous meningitis, diagnostic model, validation, non-tuberculous meningitis, nomogram, laboratory data

## Abstract

**Objective:**

We developed and validated a diagnostic scoring system for tuberculous meningitis (TBM) using 13 laboratory parameters, comparing tuberculous meningitis (TBM) and non-tuberculous meningitis (non-TBM).

**Methods:**

This study enrolled patients diagnosed with meningitis. We retrospectively collected and analyzed demographic data (gender, age) and cerebrospinal fluid (CSF) parameters, including biochemical profiles and white blood cell counts with differential analysis. Variable selection was performed using least absolute shrinkage and selection operator (LASSO) regression. The dataset was randomly divided into a training set and a validation set. A diagnostic prediction model was developed using logistic regression in the training set, with nomograms constructed to visually demonstrate the diagnostic relationships. Decision curve analysis (DCA) was employed to assess the clinical utility of the model. Finally, the diagnostic performance of the model was evaluated in the validation set.

**Results:**

A total of 254 patients with meningitis were included in this study. LASSO regression analysis identified four predictive variables: CSF glucose, CSF chloride, CSF protein and CSF mononuclear cells proportion. These parameters were incorporated into a logistic regression model, with weighted factors generating a diagnostic score. A score of ≥ 3 was suggestive of TBM with a sensitivity of 76.10% and a specificity of 84.10%, and the area under the curve (AUC) values was 0.86 (95% CI 0.81-0.91). Both calibration curves and DCA validated the robust performance of model.

**Conclusion:**

We developed and validated a clinically applicable diagnostic model for TBM using routinely available and low-cost CSF parameters. Our findings demonstrated that this scoring system provided reliable TBM diagnosis, particularly in countries and regions with limited microbial and radiological resources.

## 1 Introduction

Tuberculous meningitis (TBM) is a serious central nervous system (CNS) disease caused by Mycobacterium tuberculosis (MTB) and is characterized by high rates of disability and mortality, with more than 100,000 new cases each year (Méchaï and Bouchaud, 2019; Manyelo et al., 2021; Seddon et al., 2019). The prevalence and mortality of TBM remains high due to the burden of tuberculosis, human development index and the prevalence of HIV in various countries (Navarro-Flores et al., 2022; Wilkinson et al., 2017). Early diagnosis and Prompt treatment are crucial to effective control of TBM because they are closely related to the occurrence of adverse outcomes (Ahlawat et al., 2020). Early diagnosis of TBM is challenging because of its non-specific clinical symptoms and insufficient sensitivity of existing testing methods (Huynh et al., 2022). It is worth noting that the clinical symptoms of TBM are very similar to other diseases caused by CNS infections, such as bacterial meningitis (BM), viral meningitis (VM) and fungal meningitis (FM) (Manyelo et al., 2021). Clinical doctors need to rely on cerebrospinal fluid (CSF) examination, cerebral imaging results and even clinical experience to distinguish TBM from these diseases. CSF examination are a vital part of the diagnosis of CNS infections. Although diagnostic methods for TBM have been established, low sensitivity CSF smears and long-term culture often lead to delayed diagnosis (Ahlawat et al., 2020). Nucleic Acid Testing has been widely used for pathogen detection in CSF, however, its negative test results cannot exclude TBM (Donovan et al., 2020; Huynh et al., 2022). Therefore, this study established a simple and practical diagnostic model based on early laboratory parameters, visualized the diagnosis of TBM through nomograms, providing reference for clinical doctors to diagnose TBM from infectious meningitis in northern China.

## 2 Materials and methods

### 2.1 Patient recruitment

We conducted a retrospective study enrolling patients diagnosed with TBM, BM, VM and FM who were hospitalized at Henan Provincial Chest Hospital between January 2017 and June 2024. The study was approved by the Ethics Committee of Henan Provincial Chest Hospital.

### 2.2 Data collection

We collected demographic data (age, gender), CSF analysis results within a week of hospitalization, including biochemical profiles, white blood cell counts with differential analysis, microbial culture, modified Ziehl-Neelsen staining, Gram staining, Indian ink staining, and genetic testing results (GeneXpert Mycobacterium Tuberculosis/Rifampicin (GeneXpert MTB/RIF), Tuberculosis Deoxyribonucleic Acid (TB-DNA), Loop-mediated isothermal amplification (LAMP), Metagenomic Next-Generation Sequencing (mNGS)). All data were obtained from the patient’s electronic medical record file. Specifically, biochemical profiles included glucose (Glu), adenosine deaminase (ADA), chloride (Cl), lactate dehydrogenase (LDH) and protein (TP). White blood cell counts with differential analysis included leukocyte count (WBC), mononuclear cells count (MN), polymorphonuclear cells count (PMN), mononuclear cells proportion (MN%) and other nucleated cells proportion (Oth).

### 2.3 Diagnostic criteria

Definite TBM: Acid-fast bacilli(AFB) were observed in the CSF, MTB was cultured from CSF, or MTB was detected by commercial nucleic acid amplification test from the CSF (Marais et al., 2010).

Non-TBM includes BM, VM and FM:

BM: Gram positive bacteria were observed in the CSF, pathogenic bacteria was cultured from CSF, or pathogenic bacteria was detected by mNGS detection from CSF.

VM: The causes of bacteria, tuberculosis, fungi and noncommunicable meningitis (injury, cancer, autoimmune disorders, neurosarcoidosis) had been excluded and favorable outcome had been achieved in antiviral treatment, or virus was detected by mNGS detection from CSF.

FM: CSF positive for India ink stain or fungal culture, or fungus was detected by mNGS detection from CSF.

### 2.4 Exclusion criteria

Patients with any of the following conditions are excluded: (1) age under 14, (2) data on CSF biochemical profiles or white blood cell counts were missing, (3) If they had underwent a neurosurgical surgery within the previous month, (4) if they had more than two types of microbial infection, or (5) patients were diagnosed with probable or possible TBM (Marais et al., 2010).

### 2.5 Statistical analysis

All statistical analyses and data visualization were performed using R software (version 4.2.2). The dataset was randomly split (random seed = 1234) into a training set (70% of cases) and an internal validation set (30%) for model development and evaluation. Descriptive statistics were generated using the “descrTable” package. Continuous variables with normal distribution were presented as mean ± standard deviation (X ± S), while non-normally distributed variables were expressed as median ± interquartile range (M ± IR). The two groups comparisons were performed using independent samples t-test or Mann-Whitney U test for continuous variables, and chi-square test or Fisher’s exact test for categorical variables. Variable correlations were assessed and visualized using heatmaps generated by the “corrplot” package. Variable selection was performed using least absolute shrinkage and selection operator (LASSO) regression with the “glmnet” package, where the optimal variables within one standard error was selected to ensure model parsimony while maintaining predictive accuracy. Before logistic regression analysis, continuous variables were dichotomized at optimal diagnostic cut-off values using the “pROC” package, with only significant variables retained in the final model. The diagnostic index (DI) for each variable was calculated as the rounded β coefficient (DI = round (β)). The total diagnostic index (TDI) was derived by summing the DIs of all included variables: TDI = DI (CSF glucose) + DI (CSF chloride) + DI (CSF protein) + DI (CSF mononuclear cells proportion). The TDI served as a diagnostic tool to differentiate TBM from non-TBM cases. Calibration curves were generated using the “caret” package, and decision curve analysis (DCA) was performed with the “dcurves” package. P-value less than 0.05 was considered statistically significance.

## 3 Results

### 3.1 Basic features of the patient

Finally 254 patients admitted to Henan Chest Hospital were included in this study from January 2017 to June 2024. We diagnosed 119 cases as definite TBM and 135 cases as non-TBM (BM 61, VM 48, FM 26) according to the diagnostic criteria. Among 119 patients with TBM, 46 of 68 (67.65%) positive by CSF culture, 7 of 56 (12.50%) positive by Ziehl-Neelsen staining, 62 of 78 (79.50%) positive by GeneXpert MTB/RIF, 14 of 20 (70.00%) positive by TB-DNA, 3 of 6 (50.00%) positive by LAMP, 24 of 30 (80.00%) positive by mNGS. Patients were then randomly divided into a training set (n=180) for model development and a validation set (n=74) for internal validation. The laboratory data of the training set and validation set were compared, and there was no significant statistical difference (**Table 1**).

**Table 1:** Equilibrium test for training and validation sets.

	Total (n=254) n (%); X ± S; M ± IR	Training set (n=180) n (%); X ± S; M ± IR	Validation set (n=74) n (%); X ± S; M ± IR	*P*
Age (y)	40.05 ± 18.08	41.05 ± 18.44	37.62 ± 17.05	0.170
Male: Female (Male %)	157:97 (61.81)	114:66 (63.33)	43:31 (58.11)	0.436
Clear CSF appearance	232 (91.34)	164 (91.11)	68 (91.89)	0.841
CSF glucose (mmol/L)	2.80 ± 1.26	2.80 ± 1.26	2.82 ± 1.29	0.919
CSF adenosine deaminase (U/L)	4.97 ± 10.46	4.24 ± 5.25	6.75 ± 17.52	0.082
CSF chloride (mmol/L)	118.85 ± 9.49	118.81 ± 9.36	118.94 ± 9.87	0.924
CSF lactate dehydrogenase (U/L)	78.96 ± 180.28	67.83 ± 128.83	106.03 ± 266.25	0.125
CSF protein (mg/dL)	139.24 ± 97.12	138.73 ± 94.97	140.49 ± 102.81	0.896
CSF leukocyte count (×10^6^/mL)	132.35 ± 262.62	123.56 ± 199.65	153.72 ± 375.02	0.407
CSF mononuclear cells count (×10^6^/mL)	84.54 ± 152.34	84.83 ± 163.16	83.82 ± 123.08	0.962
CSF polymorphonuclear cells count (×10^6^/mL)	47.81 ± 170.99	38.73 ± 87.90	69.89 ± 285.81	0.187
CSF mononuclear cells proportion (%)	75.72 ± 26.53	74.52 ± 26.01	78.63 ± 27.74	0.262
CSF other nucleated cells proportion (%)	2.27 ± 11.38	1.95 ± 11.66	3.04 ± 10.72	0.492

X ± S: Mean ± Standard deviation, M ± IR: Median ± Interquartile ranges.

### 3.2 Screening variables affecting diagnosis

**Figure 1:**
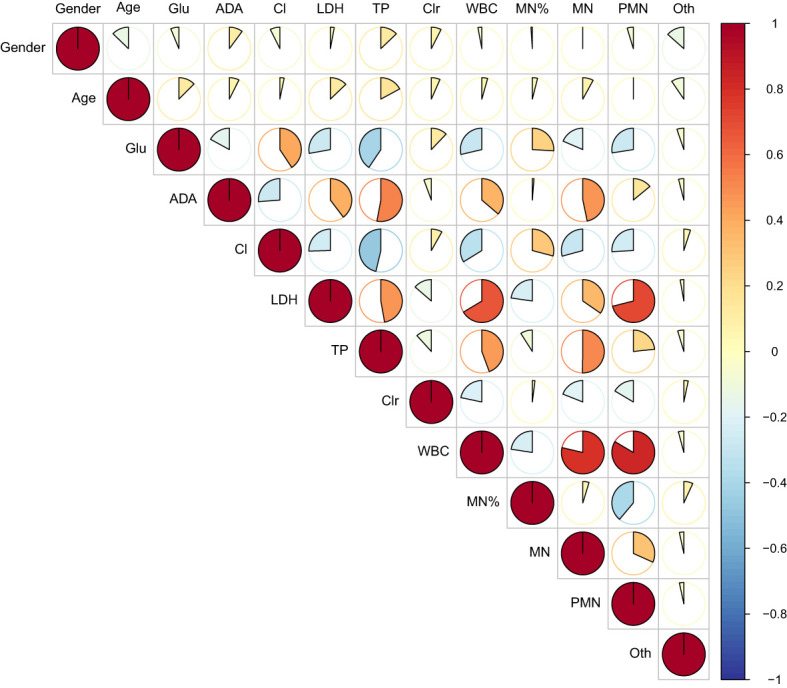
Heat map of correlations between variables. It showed that there was covariance among CSF laboratory variables.

**Figure 2:**
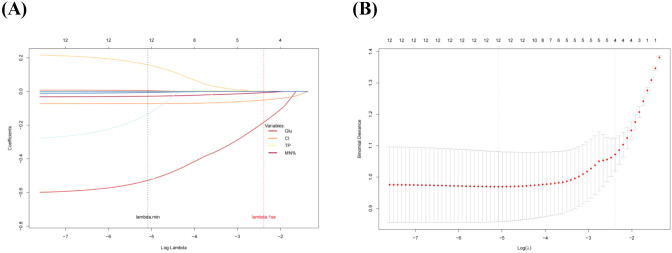
LASSO regression screening variables. **(A)**, LASSO regression selected 4 key predictors from 13 variables. **(B)**, The optimal penalty parameter (λ) was determined using the one-standard-error (lambda.1se) rule, which ensuring a robust balance between model simplicity and overfitting control.

**Figure 3:**
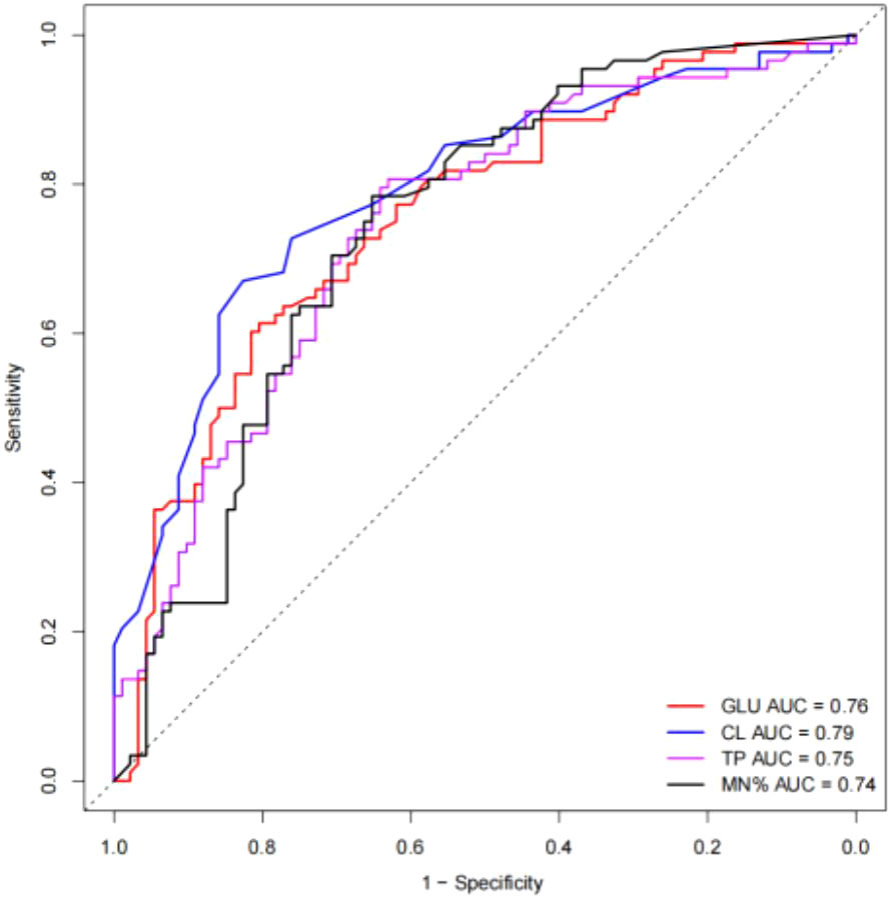
ROC curves of selected single variable on TBM diagnosis. The AUC values were all less than 0.8.

**Table 2** presented the univariate analysis of laboratory parameters between TBM and non-TBM, revealing statistically significant differences in multiple CSF parameters, including glucose, adenosine deaminase, chloride, lactate dehydrogenase, protein, leukocyte count, mononuclear cells count, polymorphonuclear cells count and mononuclear cells proportion. We subsequently observed some association among these variables (**Figure 1**). To mitigate multicollinearity among predictor variables in the model, we employed LASSO regression for variable selection and identified four optimal variables (**Figure 2**): CSF glucose, CSF chloride, CSF protein and CSF mononuclear cells proportion. We plotted these variables as receiver operating characteristic (ROC) curves, and the results were shown in **Figure 3**. Their AUC did not exceed 0.80, indicating low diagnostic performance for single variables.

**Table 2:** Comparative analysis of variables between patients with TBM and patients with non-TBM.

	TBM (n=88) n(%); X ± S; M ± IR	Non-TBM (n=92) n(%); X ± S; M ± IR	*P*
Age (y)	41.39 ± 18.73	40.73 ± 18.26	0.812
Male: Female (Male%)	55:33 (62.50)	59:33 (64.13)	0.310
Clear CSF appearance (%)	77 (87.50)	87 (94.57)	0.096
CSF glucose (mmol/L)	2.27 ± 1.08	3.30 ± 1.22	<.001
CSF adenosine deaminase (U/L)	6.50 ± 6.03	2.07 ± 3.14	<.001
CSF chloride (mmol/L)	114.10 ± 9.04	123.33 ± 7.21	<.001
CSF lactate dehydrogenase (U/L)	102.27 ± 175.39	34.89 ± 31.56	<.001
CSF protein (mg/dL)	176.93 ± 103.09	102.19 ± 69.43	<.001
CSF leukocyte count (×10^6^/mL)	194.23 ± 246.06	55.97 ± 105.23	<.001
CSF mononuclear cells count (×10^6^/mL)	121.09 ± 207.06	50.15 ± 94.28	0.003
CSF polymorphonuclear cells count (×10^6^/mL)	73.14 ± 115.32	5.82 ± 15.55	<.001
CSF mononuclear cells proportion (%)	65.12 ± 25.41	83.51 ± 23.36	<.001
CSF other nucleated cell proportion (%)	1.32 ± 2.66	2.56 ± 16.12	0.481

X ± S: Mean ± Standard deviation, M ± IR: Median ± Interquartile ranges.

### 3.3 Development of TBM diagnosis model and nomogram

**Figure 4:**
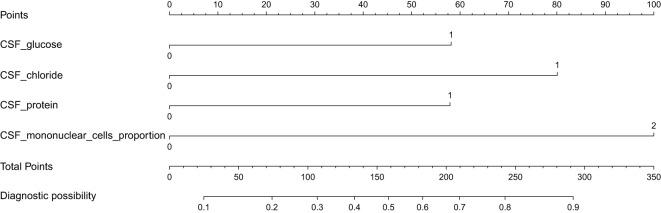
A predictive nomogram for the early diagnosis of TBM. When the total points > 158, the corresponding probability exceeds 0.5, indicating a higher likelihood of TBM.

Variables selected were modeled using logistic regression. To enhance clinical utility, continuous variables were dichotomized by the closest-to-top-left criterion in ROC curves. We used CSF glucose < 2.6 mmol/L, CSF chloride < 120 mmol/L, CSF protein ≥ 95 mg/dL, CSF mononuclear cells proportion < 87% as the cutoff points to create these dichotomous variables represented by “1”, other intervals are represented by “0”. As demonstrated in **Table 3**, all variables maintained statistical significance in the final logistic model. Diagnostic score were subsequently derived based on the β coefficients (**Table 4**). It is worth noting that in order to facilitate the application of the algorithm in clinical practice, we appropriately corrected the regression coefficient, specifically CSF glucose, CSF chloride and CSF protein were given 1 points, and CSF mononuclear cells proportion was given 2 points. The prediction model was established based on DI. TDI was calculated by summing all variables DIs. According to the ROC curve, the best cut-off point of TDI was 3. Patients with TDI ≥ 3 were classified as TBM, and patients with TDI < 3 were classified as non-TBM. The model had 76.10% sensitivity and 84.10% specificity. Finally, we drew a nomogram that can be used in clinical practice based on the logistic regression model (**Figure 4**).

**Table 3:** Multivariate logistic regression analysis about the converted variables.

	β	SE	*P*	OR (95%CI)
CSF glucose of less than 2.6 mmol/L	0.96	0.40	0.016	2.61 (1.20 ~ 5.66)
CSF chloride of less than 120 mmol/L	1.32	0.43	0.002	3.74 (1.61 ~ 8.66)
CSF protein of more than 95 mg/dL	0.95	0.44	0.031	2.59 (1.09 ~ 6.16)
CSF mononuclear cells proportion of less than 87%	1.65	0.39	<.001	5.19 (2.39 ~ 11.24)

β, β-Coefficient; SE, Standard error; OR, Odds Ratio; CI, Confidence Interval.

**Table 4:** Weighted diagnostic index (DI) scores of CSF laboratory parameters in the diagnostic rule.

variables	Diagnostic index
CSF glucose (mmol/L)	
<2.6	1
≥2.6	0
CSF chloride (mmol/L)	
<120	1
≥120	0
CSF protein (mg/dL)	
≥95	1
<95	0
CSF lymphocyte proportion (%)	
<87	2
≥87	0

### 3.4 Evaluation of diagnostic models

**Figure 5:**
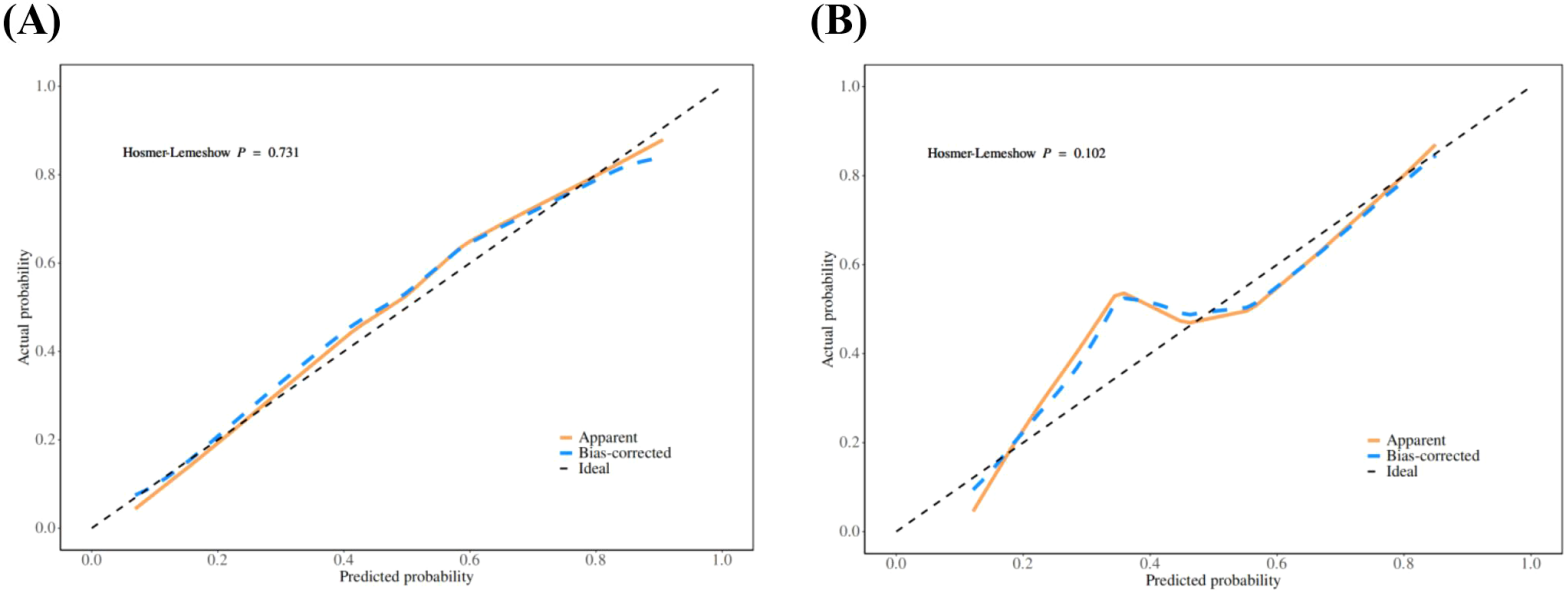
The calibration curves of training set **(A)** and validation set **(B)**. The x-axis represents the predicted probability and y-axis represents the actual probability of TBM. The reference line is 45° dashed line and indicates perfect calibration (*P >*0.05).

**Figure 6:**
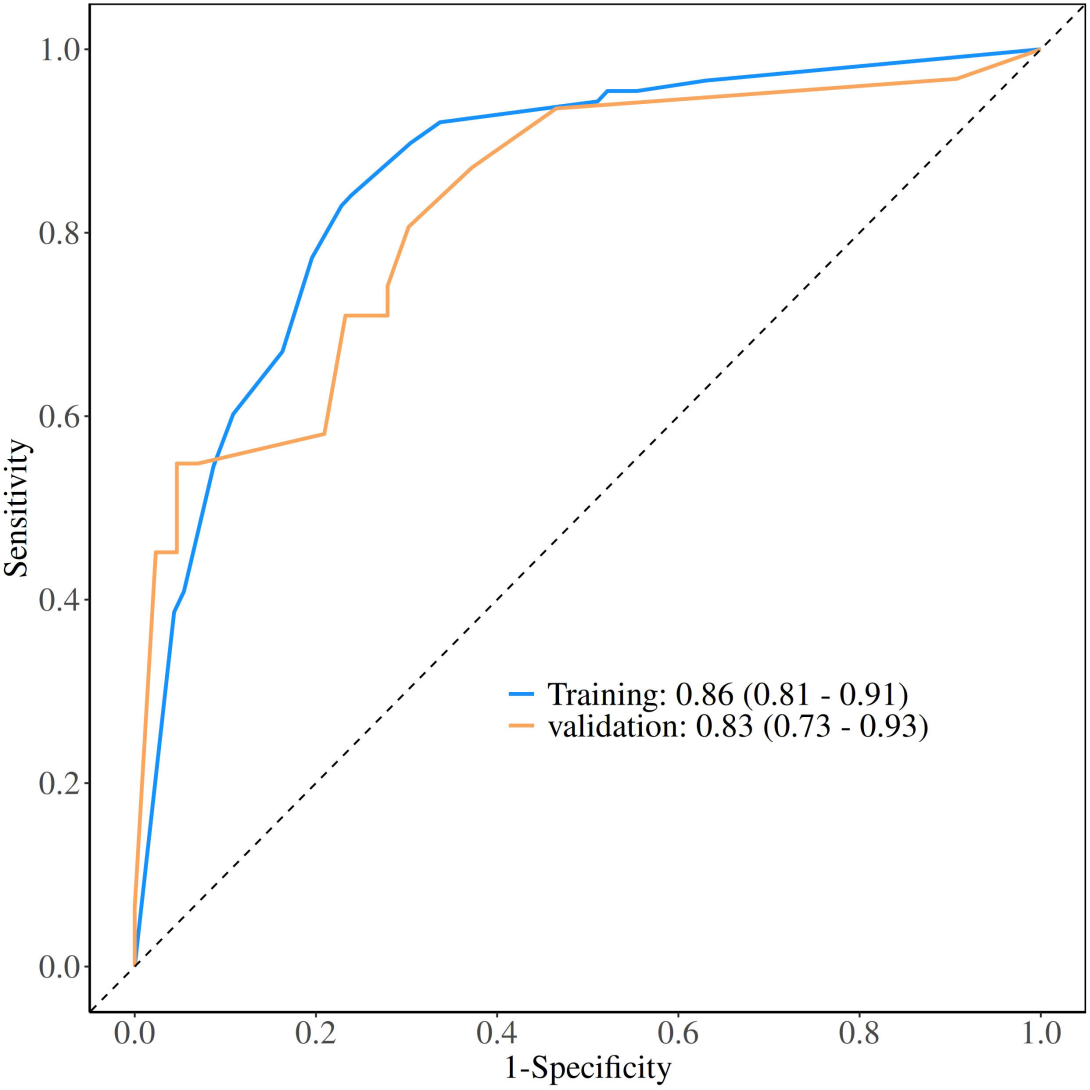
The ROC curves of training set and validation set.

**Figure 7:**
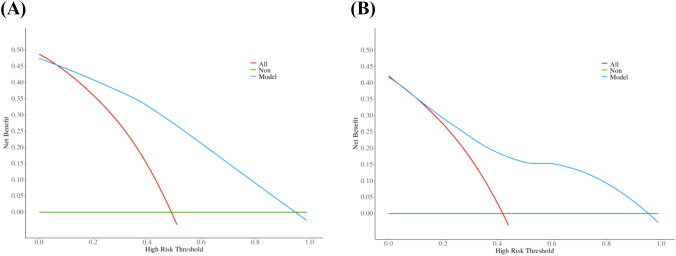
The decision curves of training set **(A)** and validation set **(B)**. The y-axis indicates the net benefit. The red line represents the hypothesis that all patients will develop TBM, and the green line represents the hypothesis that no patient will develop TBM. The model showed an obvious net benefits in both the training set and the validation set, indicating its value in clinical decision-making.

The calibration curve derived from the training set demonstrated good agreement between predicted probabilities and observed outcomes (**Figure 5A** P = 0.731). The ROC analysis demonstrated good performance with an AUC of 0.86 (95% CI 0.81-0.91)(**Figure 6**). We then evaluated the model’s diagnostic performance in the internal validation set. The model demonstrated good calibration (**Figure 5B** P = 0.102) and achieved an AUC of 0.83 in ROC analysis. The DCA curves (**Figure 7**) revealed that the nomogram demonstrated enhanced diagnostic efficacy for TBM in both the training and validation sets, indicating its clinical effectiveness.

## 4 Discussion

Despite significant progress in the global fight against tuberculosis (TB) in recent years, there is still a trend towards a reversal (Bagcchi, 2023). The morbidity and mortality remain high in many low-income countries, despite TB being a preventable and curable disease (World Health Organisation, 2021). In South-East Asia, Africa and the Western Pacific, poor socioeconomic conditions and limited medical resources not only increase the incidence of TB but also make it more difficult to control the disease (Chakaya et al., 2021; Lei et al., 2023). TBM is one of the most severe forms of TB, affecting the central nervous system (Huynh et al., 2022). Early diagnosis and timely treatment are crucial for effectively managing TBM. However, atypical clinical presentations and the low sensitivity of available laboratory tests often lead to delays in diagnosis, with the result that many patients miss the optimal treatment window (Van and Farrar, 2014). The concentration of MTB in CSF is typically low, making detection and confirmation challenging. Traditional microscopic examination and culture methods often failed to effectively diagnose TBM (Seid et al., 2023). Studies had shown that the sensitivity of CSF Ziehl-Neissen AFB smears varied considerably, ranging from 10% to 40%, and required an adequate collection (Stadelman et al., 2022; Xiang et al., 2023). Additionally, the sensitivity of CSF culture usually ranged from 50% to 70% and was time-consuming with obvious limitations (Bahr et al., 2019; Ahlawat et al., 2020). In recent years, new molecular diagnostic technologies had appeared, such as Xpert MTB/RIF and its upgraded version, Xpert Ultra, which had shown good performance (de Almeida et al., 2020). mNGS also had well sensitivity, which exceeds the sensitivity of smear microscopy and other conventional methods (Chen et al., 2022). It is important to note that these tests do not completely rule out TBM, meaning that a proportion of infections remain undetected (Donovan et al., 2020). Therefore, early diagnosis of TBM remains crucial. TBM shares many similarities with BM, VM and FM in terms of clinical presentation, making it difficult to diagnose. Firstly, the symptoms of TBM are usually non-specific including fever, headache, neck stiffness and altered consciousness, which are very similar to other types of meningitis (Donovan et al., 2019). TBM may also resemble other types of meningitis on imaging, especially in the early stages when imaging features are not clear enough for definitive diagnosis (Poplin et al., 2020). The diagnosis of TBM is a complex process involving a comprehensive assessment of clinical symptoms, imaging features and laboratory parameters, and although CSF laboratory data is only part of the diagnosis of TBM, it plays an important role (Shahan et al., 2021). This study described early laboratory data between TBM patients and non-TBM patients and developed an intuitive and simple diagnostic score of TBM based on screening important variables and rebalancing the weights of each indicator.

A total of 254 meningitis cases were included in this study, including 119 TBM patients and 135 non-TBM patients. These patients were selected based on specific inclusion criteria. Specifically, All TBM patients (probable or possible TBM was excluded) had clear pathogenic evidence and the first CSF test results at their admission were collected. Multiple CSF test results were collected in non-TBM patients within a week of determining the evidence of pathogenicity. These stringent inclusion criteria ensured the rigor of model construction. We found significant differences in CSF glucose, CSF adenosine deaminase, CSF chloride, CSF lactate dehydrogenase, CSF protein, CSF leukocyte count, CSF mononuclear cells count, CSF polymorphonuclear cells count and CSF mononuclear cells proportion between TBM patients and non-TBM patients. Because the obvious correlation between these variables, we used LASSO regression to finally determine four variables: CSF glucose, CSF chloride, CSF protein and CSF mononuclear cells proportion. In order to facilitate clinical practice, we dichotomized these variables using the best cut-off point of the ROC curve and included them in the logistic regression, and finally obtained the diagnostic score according to the coefficient transformation. TDI = DI (CSF glucose < 2.6 mmol/L) + DI (CSF chloride < 120 mmol/L) + DI (CSF protein ≥ 95 mg/dL) + DI (CSF mononuclear cells proportion < 87%). It was found that the optimum segmentation point for the TDI was 3. TBM was confirmed when TDI ≥ 3, and non-TBM was confirmed when TDI < 3.

Our results found that the CSF of TBM patients was more likely to show the characteristics of low glucose, low chlorine and high protein, which was similar to previous observations (Lu et al., 2021; Luo et al., 2021; Marais et al., 2010). However, we calculated different cut-off values, which may be due to the different population distribution or single-center dataset. In addition, we observed that the CSF mononuclear cells proportion of TBM patients was significantly lower than that of non-TBM, and the CSF mononuclear cell proportion of most non-TBM patients was more than 90%. It was worth noting that although the cytology of CSF played an indispensable role in TBM, there were not many reports about the mononuclear cells proportion in the diagnosis of TBM. Studies had indicated that a transient polymorphonuclear predominance may be observed during the first week of illness (Gupta et al., 2025), suggesting that the proportion of mononuclear cells might decrease in the acute phase. Although most studies considered CSF lymphocytic predominance (>50%) as a characteristic feature of TBM (Marais et al., 2010; Shahan et al., 2021), the cytological profile of CSF still required further verification due to variations in disease progression and methodologies used for CSF cell classification.

Our model achieved an AUC of 0.86, significantly outperforming single-variable predictors (AUC < 0.8). Consistent with accumulating evidence, reliance on a single indicator often failed to yield good diagnostic performance. To address clinical needs while minimizing testing costs, we combined some routine CSF laboratory parameters to enhance early diagnostic efficacy for TBM, particularly in resource-limited settings. Notably, our model demonstrated a sensitivity of 0.76, outperforming conventional tests and some nucleic acid tests, though slightly lower than GeneXpert MTB/RIF (79.5%) and mNGS (80.0%) which had not been widely adopted due to limitations in equipment requirements and cost. Therefore, we recommend that patients classified as TBM (TDI scores ≥ 3) should undergo further testing or be evaluated in combination with GeneXpert MTB/RIF to minimize the likelihood of missed diagnoses.

The model demonstrated good discriminative ability in the internal validation set. The calibration curves revealed strong agreement between predicted and observed risks in the training and validation sets, while DCA confirmed the clinical utility of the model as a practical decision-making tool. The Bootstrap validation (n = 1000) also demonstrated stable model performance in the training set, with the AUC ranging between 0.83 and 0.86 in 95% of resamples, though external validation remains necessary.

It should be noted that our non-TBM cohort included patients with BM, VM and FM, which enhanced the clinical applicability compared to studies focusing on a single disease population (He et al., 2021; Liu Q. et al., 2023; Wen et al., 2022). This study had several limitations that should be acknowledged. First, the retrospective design and single-center dataset may introduce selection bias and limit generalizability. External validation using multi-center datasets is warranted to confirm our findings. Second, the analysis was restricted to CSF routine laboratory parameters without incorporating clinical symptoms and imaging features, which will also be included in the next study to provide a more comprehensive diagnosis. In addition, we also noticed that the use of transcriptomic (Pan et al., 2019) and proteomic analysis (Liu Y. et al., 2023) and CRISPR sequencing technology (Baysoy et al., 2024; Cresswell et al., 2021) can effectively screen biomarkers of pathogens, which also provides an important direction for us to improve the diagnosis of TBM in the future.

## 5 Conclusion

We developed and validated a TBM diagnostic model using routine and low-cost CSF parameters. The established scoring system can be used as an effective tool for clinicians to diagnose TBM, especially in countries and regions with limited resources. However, further research is still needed to validate this diagnostic scoring system.

## Data Availability

The original contributions presented in the study are included in the article/supplementary material. Further inquiries can be directed to the corresponding authors.
